# Tip detection-antegrade dissection and re-entry method as a bailout technique for coronary perforation in chronic total occlusion intervention: a case report

**DOI:** 10.1093/ehjcr/ytag249

**Published:** 2026-04-06

**Authors:** Shun Yokota, Nobuaki Igarashi, Tomofumi Doi

**Affiliations:** Division of Cardiovascular Medicine, Department of Internal Medicine, Japanese Red Cross Kobe Hospital, 1-3-1, Wakinohamakaigandori, Chuo-ku, Kobe 651-0073, Japan; Division of Cardiovascular Medicine, Department of Internal Medicine, Japanese Red Cross Kobe Hospital, 1-3-1, Wakinohamakaigandori, Chuo-ku, Kobe 651-0073, Japan; Division of Cardiovascular Medicine, Department of Internal Medicine, Japanese Red Cross Kobe Hospital, 1-3-1, Wakinohamakaigandori, Chuo-ku, Kobe 651-0073, Japan

**Keywords:** Chronic total occlusion, Coronary intervention, AnteOwl WR intravascular ultrasonography-based antegrade dissection and re-entry, Tip detection method, Case report

## Abstract

**Background:**

Chronic total occlusion (CTO) percutaneous coronary intervention (PCI) increases the risk of complications.

**Case summary:**

In CTO-PCI of the right coronary artery, AnteOwl WR intravascular ultrasonography (AO-IVUS; Terumo Corp., Tokyo, Japan) revealed that the antegrade guidewire had entered the subintimal space just beyond the CTO entrance and created a perforation outside the vessel approximately 1 cm distal to it. As the distal part of the AO-IVUS was outside the vessel, its removal worsened the bleeding; hence, it was left in place. Tip detection-antegrade dissection and re-entry techniques were performed using a Conquest Pro 12 Sharpened Tip guidewire (Asahi Intecc Co., Ltd, Aichi, Japan). The Conquest Pro 12 Sharpened Tip guidewire successfully re-entered from the subintimal space into the true lumen immediately before the perforation site. Because the perforation and re-entry sites were adjacent to each other, a covered stent was placed, which enabled simultaneous haemostasis at the perforation site and recanalization of the CTO lesion.

**Discussion:**

Although AnteOwl WR intravascular ultrasonography (AO-IVUS)-based tip detection-antegrade dissection and re-entry is an advanced technique, we believe that it is valuable for various PCI cases.

Learning points

- Although IVUS-based ADR using the tip detection method is an advanced technique, we believe that it is useful for various PCI cases.
- The AO-IVUS-based ADR using the tip detection method is also a useful bailout technique.

## Introduction

Percutaneous coronary intervention (PCI) for chronic total occlusion (CTO) carries an increased risk of complications compared to non-CTO-PCI.^1^ CTO-PCI is associated with an increased risk of perforation, especially in older patients and women; in heavily calcified vessels and long occlusions; and with the use of rotational atherectomy, antegrade dissection/re-entry, and retrograde approaches.^2^ As coronary perforation causes haemodynamic instability, it is necessary to control bleeding into the pericardium. The first step in managing coronary perforations involves inflating a balloon to stop bleeding into the pericardium immediately after successfully passing the guidewire through the CTO lesion. Here, we report a case of antegrade dissection and re-entry (ADR) using the tip detection method to pass a guidewire through a CTO lesion as an immediate bailout technique for coronary perforation during CTO-PCI.

## Summary figure

**Figure:**
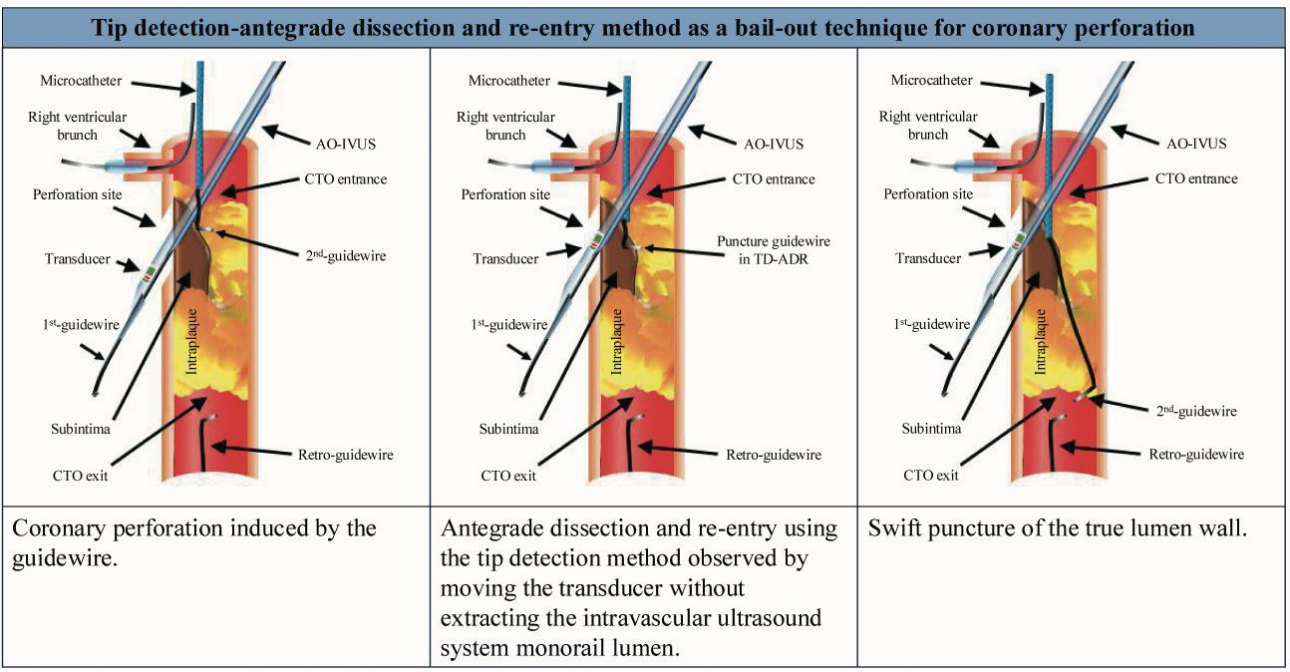


## Case presentation

**Figure 1:**
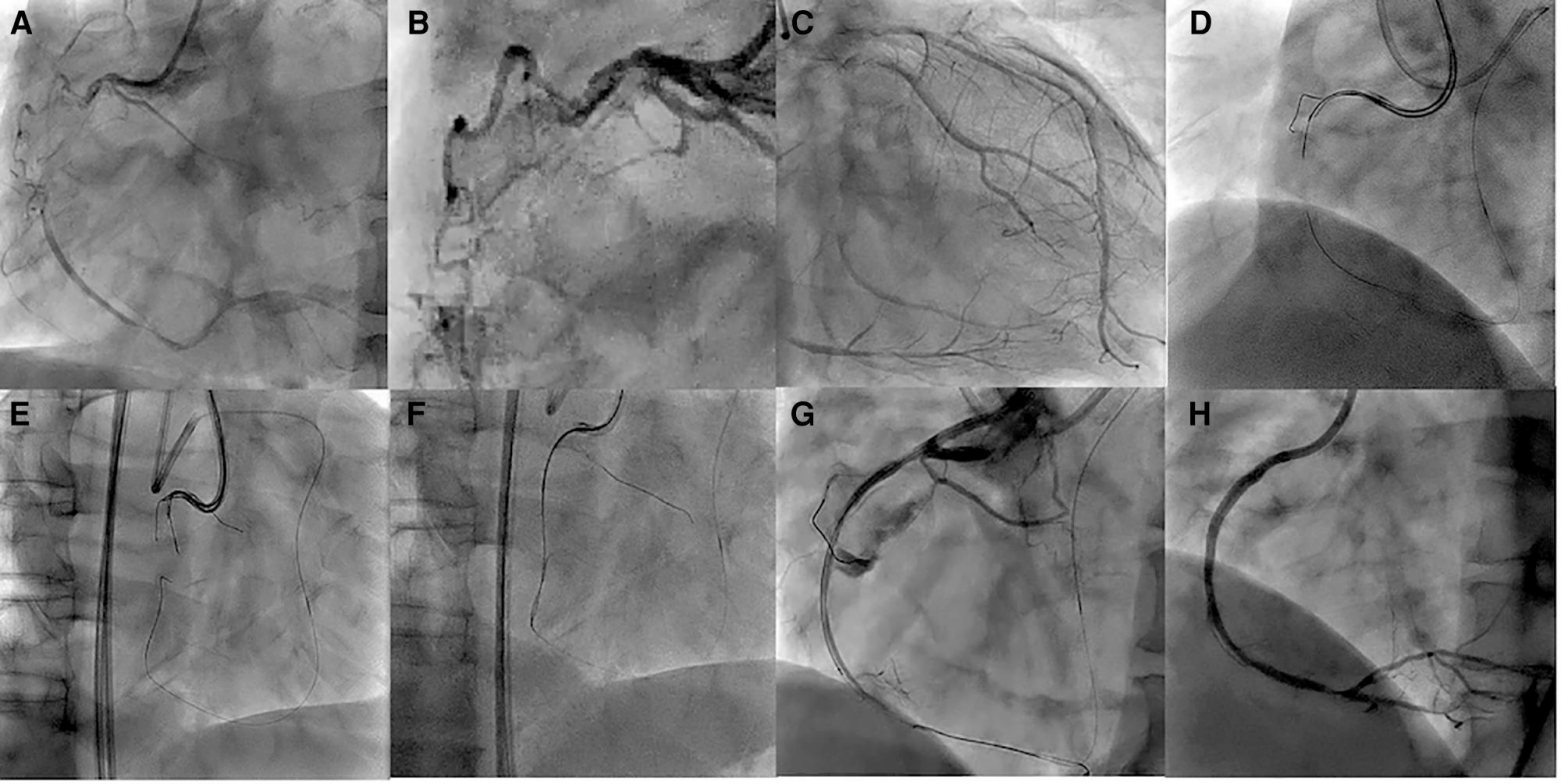
Procedures employed in the current case. *(A*, *B)* Initial angiography of the right coronary artery. *(C)* Initial angiographic image of the left coronary artery. *(D–F)* Antegrade dissection and re-entry using the tip detection method by moving the transducer without extracting the intravascular ultrasound monorail lumen. *(G)* Extravasation of contrast into the right coronary artery. *(H)* Final angiographic image of the right coronary artery.

A 49-year-old man with a history of hypertension, diabetes mellitus, and dyslipidaemia receiving optimal outpatient medical therapy underwent echocardiographic evaluation to estimate preoperative cardiovascular risk for non-cardiac surgery. Echocardiography revealed left ventricular contractile dysfunction with regional wall motion abnormality in the posterior wall (ejection fraction, 47%). Given the patient’s exertional dyspnoea, coronary angiography was performed to assess concomitant ischaemic heart disease and revealed CTO lesions in the right coronary artery (RCA), left circumflex coronary artery (LCX), and septal collaterals extending from the left anterior descending coronary artery (LAD) to the RCA (*Figure 1A–C*). We diagnosed the regional wall motion abnormality on echocardiography as being associated with exertional angina. Because an old myocardial infarction in the RCA territory was noted on electrocardiographic and echocardiographic findings, PCI of the RCA CTO was performed before the LCX CTO.

An 8-F guide catheter was inserted into the femoral artery. First, an antegrade wire escalation strategy was applied. A tapered-tip polymer-jacketed guidewire (XT-R; Asahi Intecc Co., Ltd, Aichi, Japan) supported by a Corsair microcatheter (Asahi Intecc) was unable to enter the CTO entrance at the bifurcation of the right ventricular branch. We then used moderately stiff CTO guidewires, including the Miracle Neo 3 (Asahi Intecc) and Gaia Next 2 (Asahi Intecc), also supported by Corsair. However, we could not pass the guidewires through the occluded lesion because of severe calcification at the CTO entrance. We then switched to a retrograde approach through the septal channel from the LAD. The retrograde guidewire was passed through the septal channel and advanced to the CTO exit; however, the microcatheter could not traverse the septal channel. Therefore, we performed kissing wiring, advancing the antegrade Gaia Next 2 guidewire using the retrograde guidewire as a landmark. The Gaia Next 2 guidewire was advanced towards the retrograde guidewire, and the tips of the two guidewires met in an orthogonal direction (*Figure 1D* and *E*).

**Figure 2:**
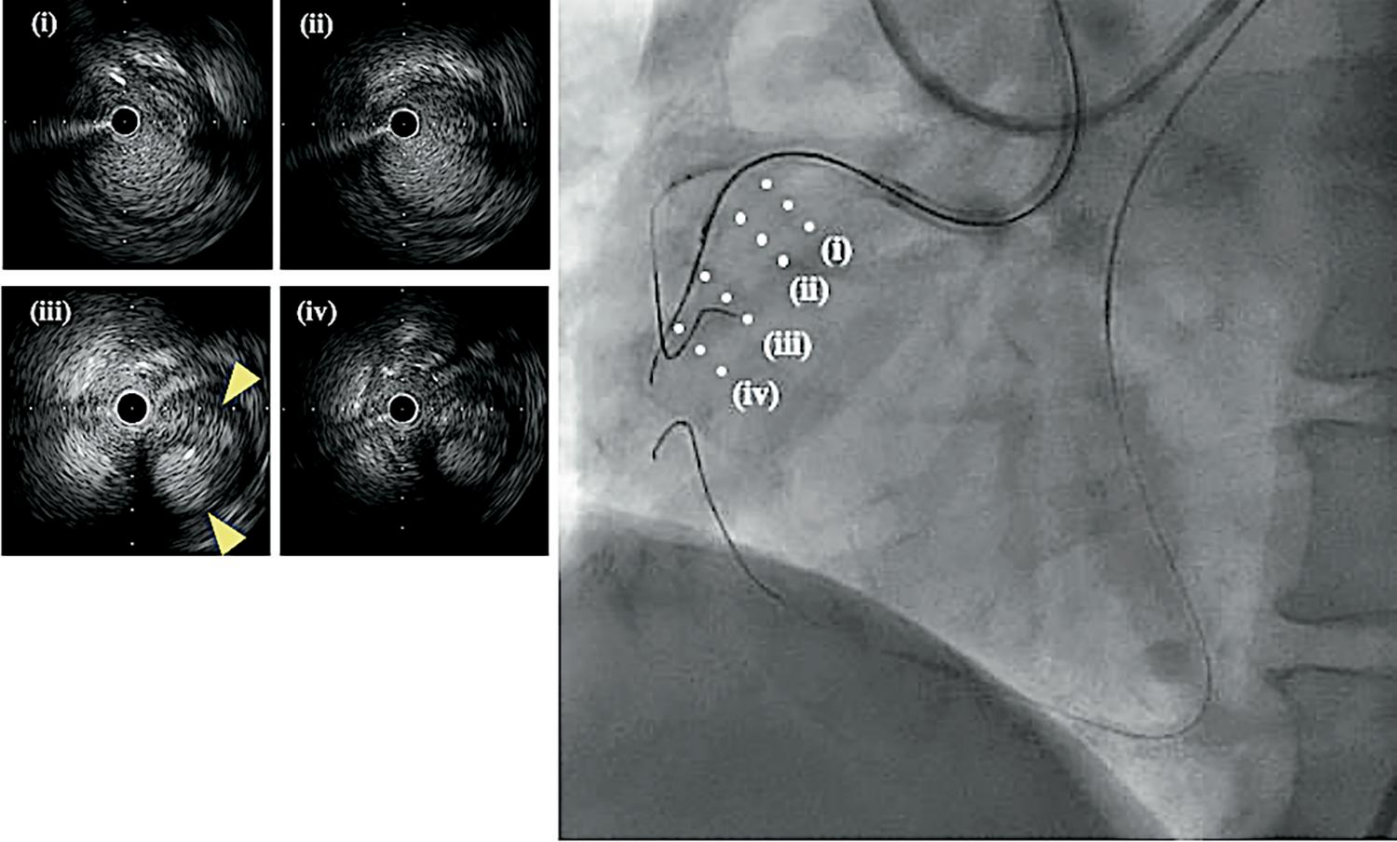
Intravascular ultrasound imaging. *(A–D)* Intravascular ultrasound images taken at each step. *(A*, *B)* Advancement of the guidewire into the true lumen. *(C*, *D)* Advancement of the guidewire into the extravascular space. The arrowheads indicate the typical appearance of a lotus root.

After the Gaia Next 2 guidewire was changed to Miracle Neo 3, AnteOwl WR, intravascular ultrasound (AO-IVUS; Terumo Corp., Tokyo, Japan) revealed that, because of severe calcification at the entrance of the CTO, the antegrade guidewire had entered the subintimal space just beyond the CTO entrance at the bifurcation with the right ventricular branch and perforated outside the vessel approximately 1 cm distal to the CTO entrance (*Figure 2*). Haemodynamics remained stable at the time of perforation, and no mechanical circulatory support was required.

**Figure 3:**
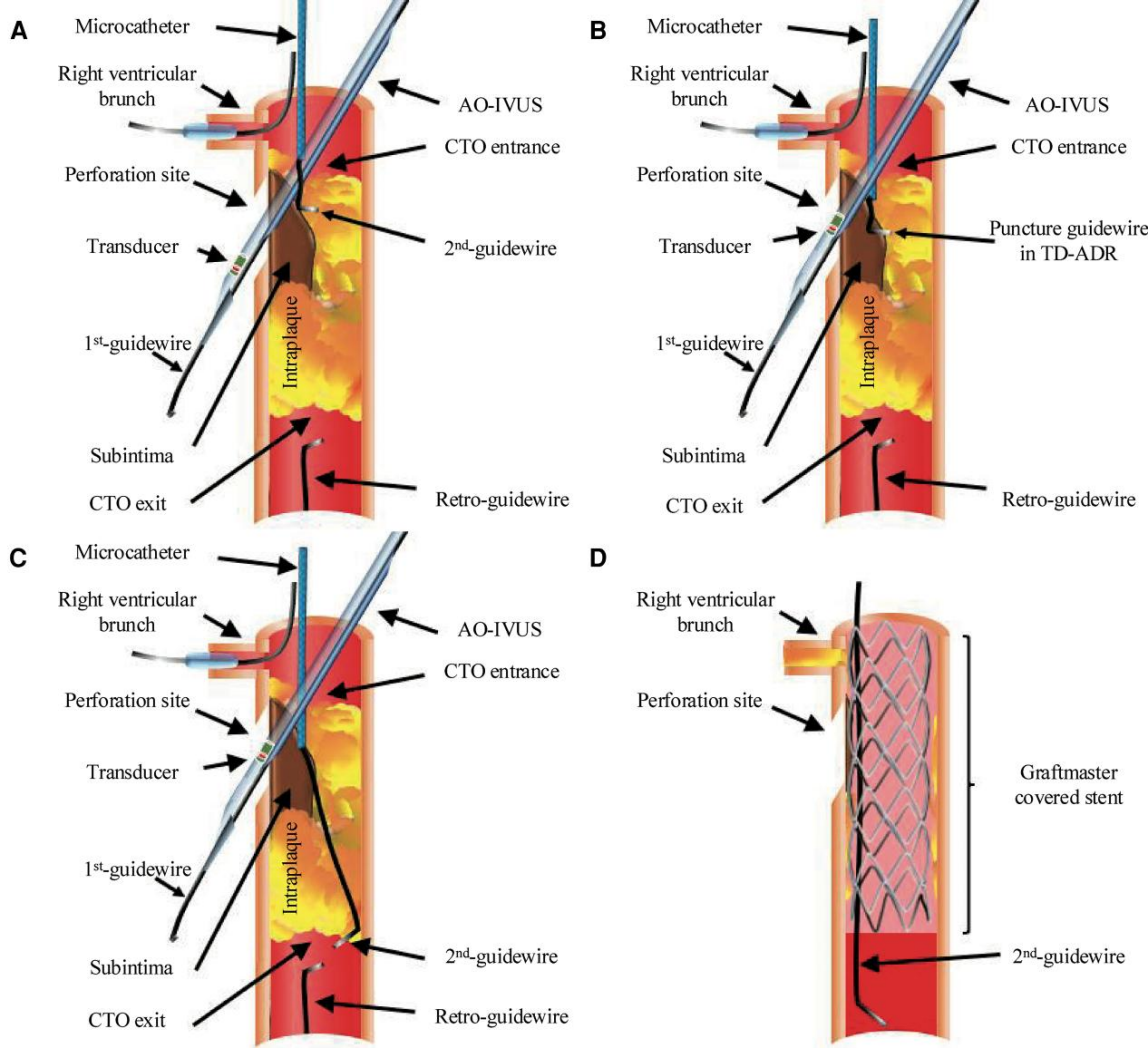
Intravascular ultrasound-based antegrade dissection and re-entry using the tip detection method. *(A)* Coronary perforation induced by the guidewire. *(B)* Antegrade dissection and re-entry using the tip detection method was observed while moving the transducer without extracting the intravascular ultrasound system monorail lumen. *(C)* Swift puncture of the true lumen wall. *(D)* The covered stent successfully sealed the perforation, but the right ventricular branch was occluded. CTO, chronic total occlusion.

During the procedure, pericardial effusion was monitored using transthoracic echocardiography; however, drainage was not performed because only a small amount of effusion was observed. Because an Ellis Grade III coronary perforation occurred upon inserting the CTO guidewire and IVUS, we left the AO-IVUS in place to stop the bleeding. AO-IVUS is a short-tip and pull-back IVUS; therefore, we could observe the area before and inside the CTO lesion by moving the transducer, while the tip of the AO-IVUS was located beyond the perforation site. Subsequently, we attempted to navigate the second wire from the subintimal space to the true lumen just before the perforation site using the tip detection-ADR (TD-ADR) technique, which simultaneously enabled haemostasis at the perforation site and recanalization of the CTO lesion. We performed TD-ADR using the Conquest Pro 12 Sharpened Tip guidewire (Asahi Intecc) supported by Corsair and succeeded in re-entry 2 mm before the perforation site (*Figure 3A* and *B*). The Corsair was then advanced into the true lumen, and the Conquest Pro 12 Sharpened Tip guidewire was changed to a soft guidewire that was advanced distally (*Figures 1F* and *3C*). Prolonged balloon inflation was unsuccessful in stopping extravasation.

Because the perforation and re-entry sites were close to each other, we determined that a covered stent was preferable to a regular stent for haemostasis. We implanted a Graftmaster (Abbott Vascular, Santa Clara, CA, USA) covered stent (2.8 mm × 16 mm). Nevertheless, this was not sufficient to stop the extravasation (*Figure 1G*). Further dilatation of the covered stent successfully sealed the perforation; however, the right ventricular branch was occluded (*Figure 3D*). Fortunately, there were no adverse sequelae after the right ventricular branch occlusion. No changes in the electrocardiography, echocardiography, and cardiac biomarkers were also noted. After successful cessation of the extravasation, a 2.75 mm × 15 mm drug-eluting stent was implanted in the distal RCA (*Figure 1H*). Consequently, PCI was performed with a target activated clotting time (ACT) of 250–300 s. Despite the coronary artery perforation during the procedure, the patient remained haemodynamically stable, allowing successful haemostasis without intraprocedural ACT reversal.

## Discussion

Here, the AO-IVUS-based TD-ADR was effective in allowing simultaneous haemostasis and recanalization by immediately passing a guidewire through the CTO lesion and implanting a covered stent on the coronary perforation without resultant haemodynamic instability. Because CTO-PCI carries an increased risk of complications compared to non-CTO-PCI,^1^ it is important to have sufficient knowledge to deal with the various complications that might occur. The first step in managing coronary perforation in CTO-PCI is to inflate the balloon to stop bleeding into the pericardium and determine whether a conventional or covered stent needs to be implanted. In this case, the alternative therapeutic option was to re-establish the retrograde approach; however, tip detection and re-entry (TD-ADR) was performed because reverse-controlled antegrade and retrograde tracking (CART) (reverse CART) carried a potential risk of occluding the sinus node artery, and the retrograde guidewire could not be advanced into the antegrade lumen. Moreover, endovascular coiling using the antegrade approach was not performed because it would prevent a re-attempt of PCI.

To achieve haemostasis and recanalization simultaneously, we should quickly pass a guidewire through the CTO lesion, and ADR is one of the strategies in the ‘hybrid algorithm’ for CTO crossing.^3^ Crossing and re-entry systems, such as the CrossBoss catheter and Stingray re-entry system (BridgePoint Medical System, BridgePoint Medical, Plymouth, MN, USA), have been developed to simplify and potentially improve successful antegrade recanalization.^4^ Current device-based ADR systems such as Stingray-ADR have been widely used; however, they lack a high level of accuracy because they rely on angiographic observations without IVUS.^5^ Recently, AO-IVUS-based TD-ADR has shown efficacy in CTO-PCI by providing real-time visualization of the target lumen and guidewire tip, thus enabling precise puncture of the distal true lumen.^6^ Furthermore, a study showed that the success rate of the ADR procedure was significantly improved and the total procedural time was significantly reduced in the TD-ADR group compared to that in the Stingray-ADR group.^5^ Although there are some issues with the long tip, poor pushability, and poor image quality of OptiCross IVUS (Boston Scientific), some studies have reported that TD-ADR can also be performed with OptiCross IVUS without using AO-IVUS.^7,8^

TD-ADR is a useful technique for quickly navigating a guidewire from the subintimal space to the true lumen. We believe that the TD-ADR is a useful technique for various cases of coronary artery occlusion during PCI, including the present case.

In conclusion, IVUS-based TD-ADR is an effective bailout technique for coronary artery perforation in CTO-PCI because it allows simultaneous haemostasis and recanalization by immediately passing a guidewire through the CTO lesion and implanting a stent.

## Data Availability

Data pertaining to this article can be shared upon reasonable request from the corresponding author.

## Lead author biography

After graduating from university in 2012, Shun Yokota entered the field of cardiology following 2 years of residency. After studying general cardiology for 3 years, he has engaged in research activities focusing on echocardiography for 4 years at Kobe University.
